# Taurine induces autophagy and inhibits oxidative stress in mice Leydig cells

**DOI:** 10.5935/1518-0557.20190079

**Published:** 2020

**Authors:** Shokofeh Yahyavy, Armita Valizadeh, Ghasem Saki, Layasadat Khorsandi

**Affiliations:** 1 Cellular and Molecular Research Center, Ahvaz Jundishapur University of Medical Sciences, Ahvaz, Iran; 2 Department of Anatomical Sciences, Faculty of Medicine, Ahvaz Jundishapur University of Medical Sciences, Ahvaz, Iran

**Keywords:** Leydig cells, taurine, autophagy, oxidative stress, testosterone

## Abstract

**Objectives:**

This study evaluated taurine (TAU) effects on autophagy, apoptosis and oxidative stress in mice Leydig TM3 cells.

**Methods:**

We treated TM3 cells with TAU (100 µg/mL) or 3-Methyladenine (3-MA, an autophagy inhibitor) for 24 h, and assessed cell viability, testosterone level, oxidative stress, apoptosis, and autophagy.

**Results:**

The results showed that TAU markedly increased cell viability, testosterone levels, expression of autophagy-related genes and percentage of LC3-II-positive cells. TAU significantly reduced malondialdehyde (MDA) contents and reactive oxygen species (ROS) levels and increased the activities of SOD (superoxide dismutase) and CAT (Catalase) enzymes in the TM3 cells. TAU in the presence of autophagy inhibitor (3-MA) increased oxidative stress and decreased testosterone levels.

**Conclusion:**

The results showed that autophagy might be involved in TAU-increased testosterone levels in mice Leydig TM3 cells.

## INTRODUCTION

Taurine (TAU) is a free amino acid that is present in several mammalian tissues (De Luca *et al.*, 2015; Park *et al*., 2002; Yang *et al*., 2010). TAU is present in some foodstuff, such as fish and meat and often added to energy drinks. TAU has several physiological functions including energy storage, membrane stabilization, antioxidation and xenobiotic conjugation (Huxtable, 1992). In male reproductive organs, TAU is present in Leydig cells, and in some other cells (Yang *et al*., 2010). Lobo *et al*. (2000) reported that TAU stimulated testosterone secretion *in vivo* and *in vitro*. Despite its importance, we still do not know the impact of TAU in Leydig cells.

Leydig cells produce testosterone in testicular tissues, where autophagy is active (Yi & Tang, 1999). In autophagy, microtubule-associated protein-1 light chain (LC3-I) converts to a lipid form, termed LC3-II, by a complex of Atg5, Atg12, and Atg16 proteins to induce autophagosome. Mammalian rapamycin (mTOR) regulates autophagy by activating several autophagy-related proteins, including Beclin-1, Atg1, Atg5, and Atg7 (He & Klionsky, 2009). Autophagy regulates ROS buildup by increasing the clearance of damaged organelles and activating antioxidant enzymes in the Leydig cells (Li *et al*., 2011).

In the present study we investigated the TAU effect on oxidative stress, autophagy, apoptosis, and testosterone level in the mouse Leydig TM3 cells.

## MATERIALS AND METHODS

### Experimental design

**Figure 1:**
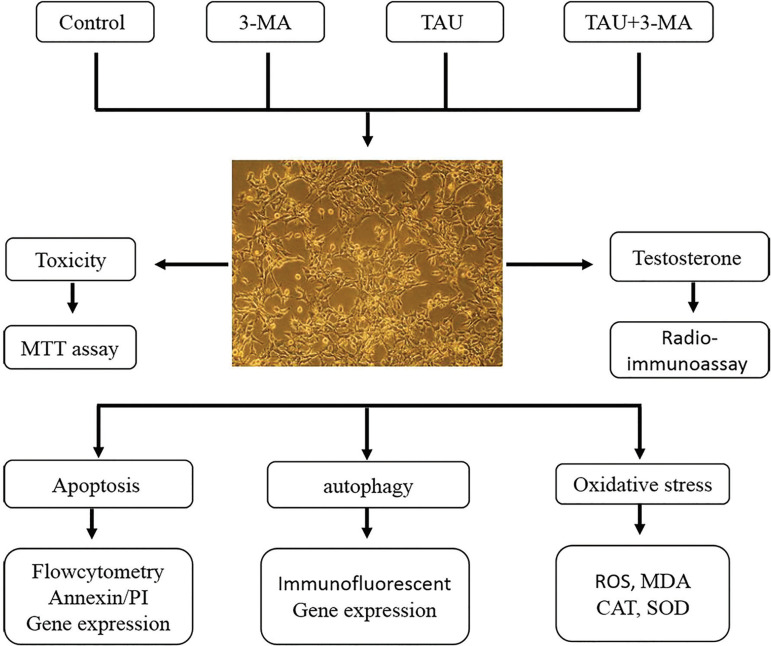
Schematic illustration of the study design

The mice Leydig TM3 cell line was bought through the National Center for Genetic and Biological Reserves in Iran*.* We cultured the cells in DMEM medium, complemented with 10% fetal bovine serum and 1% penicillin/streptomycin at 37ºC within a moist atmosphere with 5% CO_2_. The cells were incubated with TAU (Sigma) at various concentrations for 12, 24, 48 and 72h, as a pilot study (Table 1). The cells were grouped into 4 categories (Figure 1):

**Table 1:** Viability percentage in TM3 cells at different doses and duration times of TAU treatment (pilot study).

Treatments	12 h	24 h	48	72
12.5µg/mL	99.4 ± 4.5	98.7 ± 4.7	98.6 ± 2.2	100.4 ± 3.2
25 µg/mL	98.3 ± 4.8	101.5 ± 3.2	102.2 ± 5.3	102.6 ± 5.1
50 µg/mL	99.2 ± 3.5	105.7 ± 6.9	105.6 ± 4.2	104.3 ± 6.4
100 µg/mL	98.6 ± 5.5	135.2 ± 11.2*	138.1 ± 9.3*	132.8 ± 9.8*
150 µg/mL	98.8 ± 4.3	136.4 ± 12.2*	137.3 ± 10.7*	134.6 ± 12.3*
200 µg/mL	99.2 ± 3.6	136.7 ± 10.7*	137.8 ± 11.3*	135.1 ± 10.7*

Values are expressed as mean ± SD (n=6).

* *p*<0.05 *symbol indicates comparison to 12h in each row

- Control: received only media for 24h
- 3-MA (3-Methyladenine, an autophagy inhibitor): received 10 mmol 3-MA for 24h
- TAU: received 100 µg/mL TAU for 24h
- TAU + 3-MA: received 10 mmol 3-MA and 100 µg/mL TAU for 24h

### Cell viability

We cultured the TM3 (1 × 10^4^ cells/well) cells in 96-well plates. After treating them, the supernatants were disposed of and we added 20 µL of MTT solution (5 mg/ml) to all of the wells and incubated for four hours at 37ºC. Upon completion of incubation, the medium was eliminated, and we added 100 µL of DMSO to all of the wells. We used a micro-plate reader (BioRad, USA) to measure absorbance at 570 nm.

### Testosterone assay

We incubated the cell suspensions with TAU or 3-MA in the presence of hCG (0.05IU/ml) at 37ºC for 24h. We measured the testosterone concentration in culture media via a radio-immunoassay, complying with the manufacturer`s guidelines (Monobind, USA).

### Annexin V-FITC/propidium iodide apoptosis assay

We seeded the TM3 cells (1.0×10^6^/mL) in a six-well culture plate, and normal, apoptotic and necrotic cells were distinguished using an Annexin V-FITC/propidium iodide assay kit (Ebioscience™ Annexin V Kit, USA) according to the manufacturer’s instruction. We analyzed the samples using a flow cytometer (BD Facscalibur, USA). We analyzed the data using the FlowJo V10.

### Immunocytochemistry

After treating the TM3 cells, we deposited them over a glass coverslip and let it stabilize for fifteen minutes in a 4% para-formaldehyde in PBS at 4ºC. Then, we washed them in PBS. We used anti-LC3-II antibody (sc-16755, Santa Cruz) at 1/100 dilution overnight at 4ºC. After washing twice in PBS, we incubated the section with FITC-conjugated anti-mice secondary anti-body (sc-2356, Santa Cruz) for one hour at room temperature. The fluorescence pictures were captured by a fluorescence microscope (Olympus, Japan).

### Real-Time Polymerase Chain Reaction

The whole RNA was drawn out from the cells via the RNeasy Mini kit (Qiagen) in accordance with the Company's guidelines. We synthesized cDNA using a cDNA synthesis kit from total RNA regarding the manufacturer's instruction. About 2 µL of cDNA boosted in each 25 µL PCR reaction mix, which contains 10.1 µl DEPC water, 0.2 µL of each 10pM forward and reverse primers, and 12.5 µL of 2x SYBR Green Master Mix [Fermentas, Canada] (Table 2). PCR was amplified in forty cycles through this protocol: 95ºC for ten minutes, 95ºC for fifteen seconds, 95ºC for thirty seconds, and 60ºC for thirty-four seconds. We used the 2^ΔΔCT^ method to analyze the data.

**Table 2:** Primer sequences.

Groups	Forward	Reverse
** Bax **	GCTGGACATTGGACTTCCTC	ACCACTGTGACCTGCTCCA
** Bcl-2 **	GGATGCCTTTGTGGAACTGT	TCACTTGTGGCCCAGATAGG
** Beclin-1 **	CGGTTTTTCTGGGACAACAA	AAAAACGTGTCTCGCCTTTC
** mTOR **	TATTCACCTCCTGCCTCACC	TGTGATGGCTGTGAAGATCC
** Atg5 **	CCAGAAAAAGACCTTCTGCACT	CAATCCCATCCAGAGTTGCT
** LC3-II **	GATAATCAGACGGCGCTTGC	ACTTCGGAGATGGGAGTGGA
** GAPDH **	ACCCAGAAGACTGTGGATGG	TTCTAGACGGCAGGTCAGGT

### Determining MDA content, ROS level and anti-oxidant enzyme activity

We treated the TM3 cells with TAU or 3-MA for 24h. Afterwards, we performed the lysis of the gathered specimens, and the protein contents of the TM3 cells we identified via a BCA protein assay kit (Pierce Biotechnology Inc. IL). Upon having the cell lysates centrifuged, we assessed the contents of malondialdehyde (MDA), Catalase (CAT), and super-oxide dismutase (SOD) activity on the basis of the related guidelines (ZellBio, GmbH, Germany). With regards to the Company's guidelines, we measured the reactive oxygen species (ROS) using a dichloro-dihydrofluorescein diacetate (DCFH-DA) determination kit (Sigma, St. Louis, MO). We measured the ROS via a spectrofluorometer (LS50B, Waltham, USA; Ex: 490 nm, Em: 570 nm).

### Statistical analyses

We used the ANOVA SPSS 21.0 (Chicago, IL, *USA*) to analyze the data. Then, we carried out a post-hoc pair-wise comparison using the Bonferroni technique. We considered the *p* value ˂0.05 as statistically significant. Each experiment were done in quadruplicates.

## RESULTS

### Cell viability

**Figure 2:**
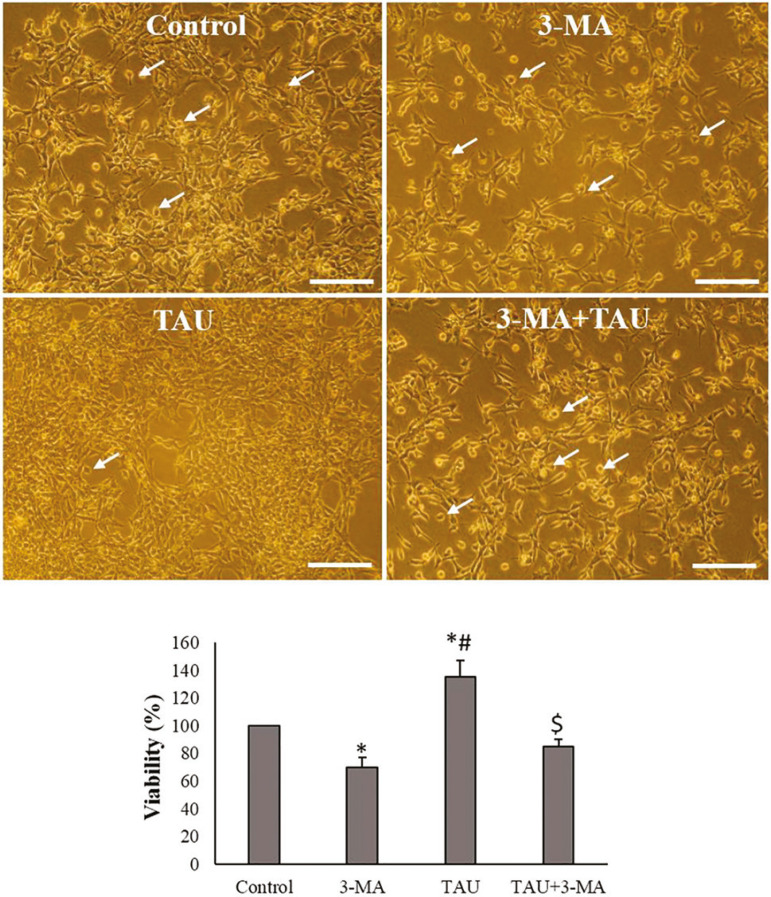
Morphology (above images) and viability (the following pictures) of TM3 cells in experimental and control groups (Scale bar: 100 µm). The arrows indicate apoptotic morphology. The values are expressed as mean ± SD. *p<0.05, ^#^ *p*<0.05, ^$^ *p*<0.05; *, ^#^ and the ^$^ symbols respectively indicate comparison to the control, 3-MA and TAU groups.

The cell viability percentage significantly decreased in 3-MA exposed TM3 cells, compared to the control cells (*p*<0.05). The cell viability significantly increased in TAU-treated cells, compared to the control cells (*p*<0.05). In the TAU + 3-MA group, the cell viability of TM3 cells significantly decreased in comparison with the TAU and 3-MA groups (Figure 2).

### Testosterone assay

**Figure 3:**
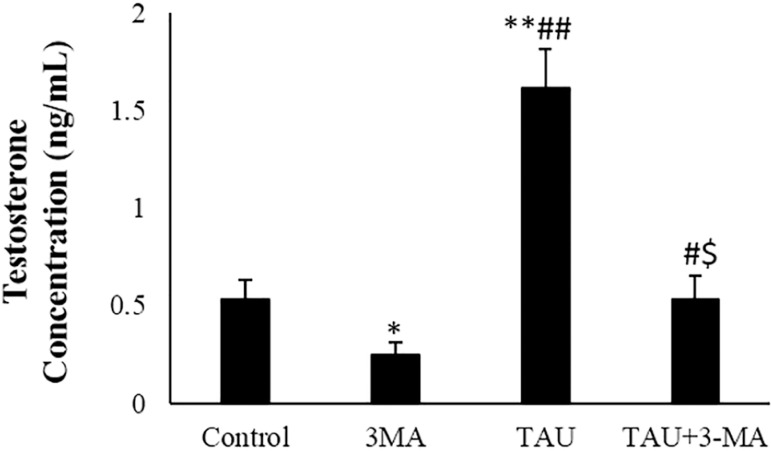
Testosterone concentration in the control and experimental groups. The arrows indicate apoptotic morphology. The values are expressed as mean ± SD. *p<0.05, **P<0.01, ^#^ *p*<0.05, ^##^ *p*<0.01, ^$^ *p*<0.05; *, ^#^ and the ^$^ symbols respectively indicates comparison to the control, 3-MA and TAU groups.

The testosterone level significantly decreased in 3-MA exposed TM3 cells when compared to the control cells (*p*<0.05). The testosterone concentration significantly increased in TAU-treated cells, compared to the control and 3-MA groups (*p*<0.01). In the TAU + 3-MA group, the testosterone level of the TM3 cells significantly decreased in comparison with the TAU-treated cells (Figure 3).

### Annexin V-FITC/propidium iodide apoptosis assay

**Figure 4:**
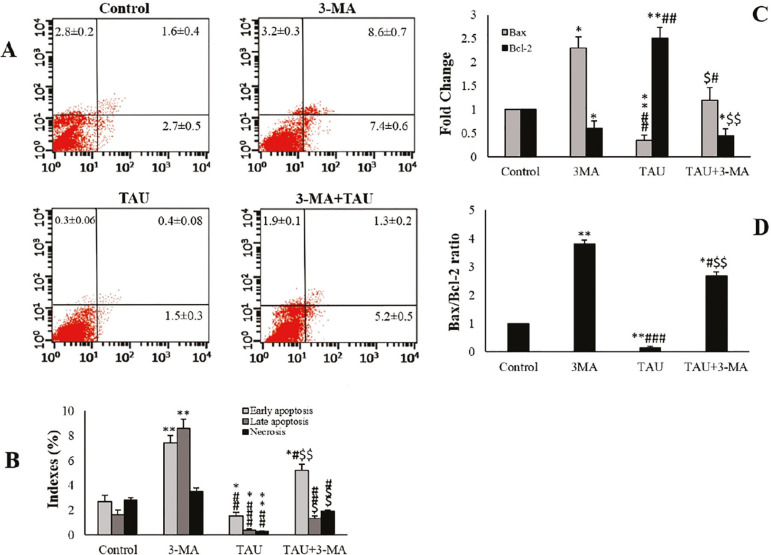
Flow cytometry of Annexin / PI staining in the experimental & control groups. Lower left quadrant: live cells; Lower right quadrant: initial apoptosis; Upper right quadrant: late apoptosis; Upper left quadrant: necrotic cells (A and B). The relative level of mRNA expression of *Bax* and *Bcl*-*2* genes and *Bax*/*Bcl*-*2* ratio (C and D). The values are expressed as mean±SD. *p<0.05, **p<0.01, ^#^ *p*<0.05, ^##^ *p*<0.01, ^###^ *p*<0.001, ^$^ *p*<0.05, ^$$^ *p*<0.01; *, ^#^ and the ^$^ symbols respectively indicate comparison to the control, 3-MA and TAU groups.

In 3-MA exposed TM3 cells, necrotic and apoptotic indices were significantly higher than those from control cells. TAU decreased the late apoptosis, necrosis, and early apoptosis percentages in TM3 cells in comparison with the control and 3-MA groups (*p*<0.01). In the TAU + 3-MA group, the apoptotic and necrotic indices significantly increased in comparison with TAU-treated cells (Figure 4), and significantly reduced when compared to the 3-MA-treated cells.

### Quantitative Real-time RT-PCR

In 3-MA exposed TM3 cells, the expression of *Bax* and *Bcl-2* considerably changed in comparison to the control group. In the TAU-treated cells, the expression of the *Bax* was considerably higher, while the expression of the *Bcl-2* significantly diminished compared to the control and 3-MA groups. There was a significant increase in the expression of the *Bax* gene while there was a significant reduction in the expression of the *Bcl*-2 gene in TAU+3-MA-treated cells, compared with the controls and TAU-treated cells (Figure 4). In 3-MA exposed TM3 cells, the *Bax*/*Bcl-2* ratio significantly increased in comparison with the control group. In the TAU-treated cells, the *Bax*/*Bcl-2* ratio significantly decreased compared to the control and 3-MA groups. The *Bax*/*Bcl-2* ratio significantly increased, compared with the control and TAU-treated cells (Figure 4).

**Figure 5:**
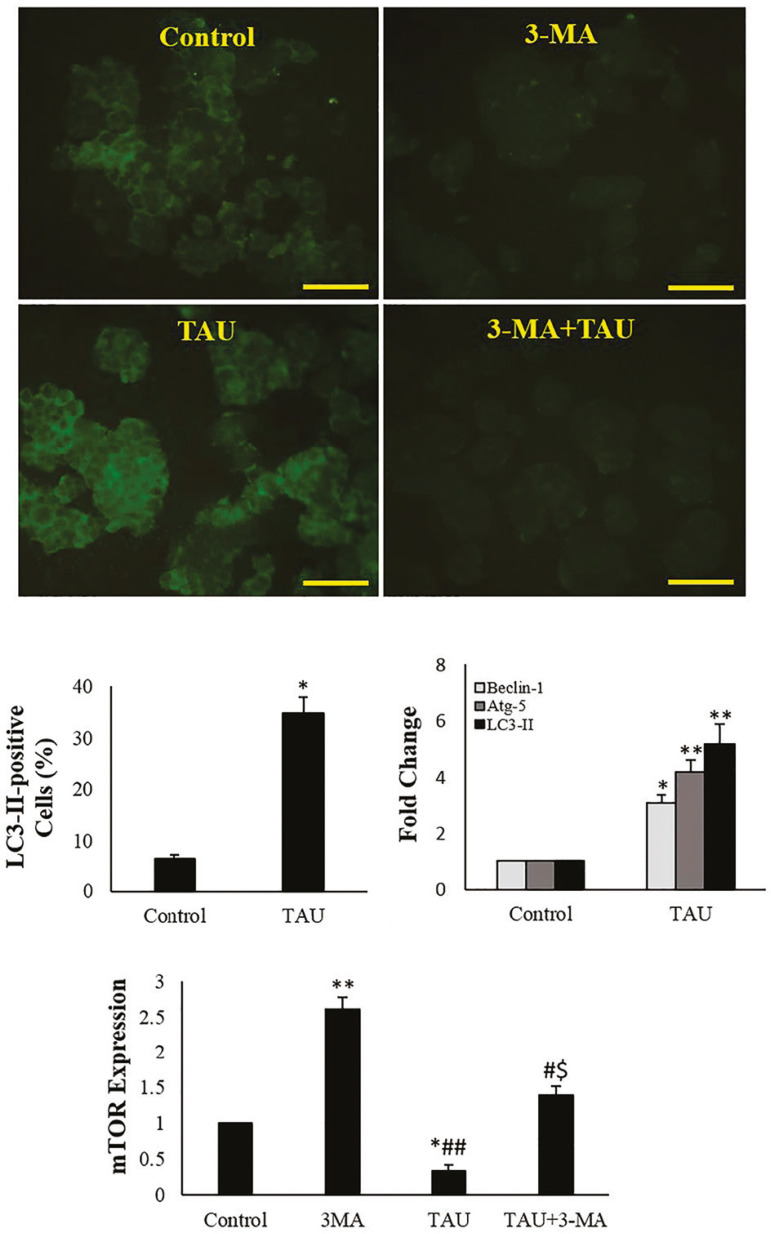
Immunofluorescent microscopy of the TM3 cells, percentage of LC3-II-positive cells and expression of autophagy-related genes. The bright green staining indicates LC3-II-positive cells (scale bars: 100 µm). The values are expressed as mean±SD; *p<0.05, **p<0.01, ^#^*p*<0.05, ^##^*p*<0.01, ^$^*p*<0.05; *, ^#^ and the ^$^ symbols respectively indicate comparison to the control, 3-MA and TAU groups.

In 3-MA-treated cells, the *LC3-II*, *Atg5* and *Beclin1* genes were not expressed while the mTOR expression was significantly higher. In TAU-treated cells, the mTOR gene expression was significantly reduced while the expression of the *LC3-II, Atg5* and *Beclin1* genes experienced a significant increase, compared to that of control cells. In TAU+3-MA-treated cells, the mTOR expression showed a significant increase in comparison with those from the TAU-treated cells. While there was a significant reduction in mTOR expression in comparison with 3-MA-treated cells (Figure 5).

### Immunocytochemistry

In the 3-MA exposed TM3 cells, we did not detect LC3-II. There was a significant increase of the percentage of LC3-II-positive cells in comparison with control group (*p*<0.05) by TAU (*p*<0.05). Figure 5 depicts these results.

### ROS levels, MDA content and antioxidant enzyme activity

**Figure 6:**
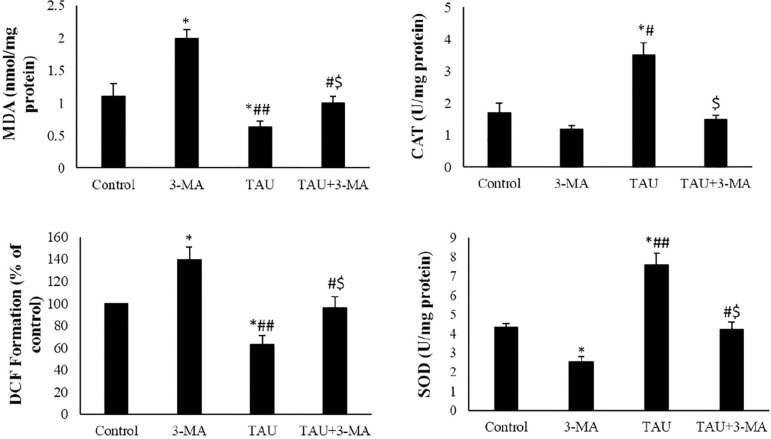
MDA contents, levels of ROS (DCF formations) and activities of the anti-oxidant enzyme in the experimental and control groups. The values were expressed as mean ±SD. *p<0.05, ^#^*p*<0.05, ^##^*p*<0.01, ^$^*p*<0.05; *, ^#^ and the ^$^ symbols respectively indicate comparison to the control, 3-MA and TAU groups

ROS level and MDA content of 3-MA exposed cells were significantly increased in comparison with those from the control group. There was a considerable reduction in MDA contents and levels of ROS in the TAU-treated cells when compared to the 3-MA and control groups (*p*<0.05). In the TAU+3-MA, the ROS level and MDA content significantly changed in comparison with the TAU and 3-MA groups. In the 3-MA exposed cells, CAT and SOD activity significantly decreased in comparison with the control group. In the TAU-treated cells, the antioxidant activity significantly increased in comparison with the 3-MA and control groups (*p*<0.05). In the TAU+3-MA, the CAT and SOD activity significantly changed in comparison with the TAU and 3-MA groups (Figure 6).

## DISCUSSION

The present study showed that TAU increased the levels of testosterone, improved antioxidant activity, decreased ROS levels, inhibited apoptosis, and induced autophagy in TM3 Leydig cells. The results show that TAU could increase the viability percentage of TM3 cells. The increasing viability may be associated with the increasing survival of TM3, but not for the proliferation of these cells. Indeed, flow cytometry and morphology evaluations showed that TAU markedly reduced apoptosis in TM3 cells. TAU decreased the Bax/Bcl-2 ratio in the TM3 cells, also showing suppression of apoptosis. Previous studies have also reported anti-apoptotic effects of TAU in noncancerous cells (Takatani *et al*., 2004; Kim *et al*., 2009; Das *et al*., 2012). Aly & Khafagy (2014) showed anti-apoptotic effects of TAU against endosulfan in adult rat testicles. TAU inhibited apoptosis in Thiopurine-induced testicular damages in rats (Ramadan *et al*., 2018). The expression of autophagy-related genes including Beclin-1, Atg5, and LC3-II was markedly increased in the TAU-treated cells. The percentage of LC3-II-positive cells also increased in the TAU-treated cells. Kaneko *et al*. (2018) reported that TAU could induce autophagy in adipocytes.

In the presence of 3-MA, TAU increased the Bax/Bcl-2 ratio in comparison with the absence of 3-MA, indicating that TAU may suppress apoptosis by enhancing the autophagy process. 3-MA in the presence of TAU decreased the apoptotic index of TM3 cells, in comparison with the absence of TAU. This finding indicated that TAU could also suppress apoptosis by involving multiple signaling pathways.

In the current study, testosterone levels dramatically increased in the TAU-treated cells, which may be associated with the increasing viability of TM3 cells or the increasing biosynthesis of androgens. Yang *et al*. (2010) showed that TAU could stimulate testosterone generation in cultured Leydig cells. The reducing testosterone level in 3-MA- treated cells indicated autophagy involved in testosterone production in the TM3 cells. Rapamycin (autophagy stimulator) induced an increase in testosterone concentration in rat Leydig cells, whereas 3-MA had the opposite effect (Yang *et al*., 2017).

Gao *et al*. (2018) revealed that autophagy increased cholesterol absorption by the Leydig cells and dysfunction of autophagy leads to insufficient testosterone production. Autophagy activity decreased in aged rat Leydig cells (Li *et al*., 2011), and testosterone levels reduced in autophagy deficiency in mice (Yoshii *et al*., 2016). The autophagy inhibition was accompanied by a massive loss of germ cells (González *et al*., 2018). By contrast, Zhang *et al*. (2012) showed that heat exposure caused autophagy-induced germ cell death and spermatogenesis defects in mice. In presence of TAU, 3-MA induced a marked increase in testosterone levels in comparison with the absence of TAU. Thus, other mechanisms may also activate testosterone production by TAU.

TAU increases testosterone levels and raises testicular antioxidation in diabetic rats (Liu *et al*., 2017). In the present study, TAU suppressed oxidative stress by reducing MDA content and ROS levels, and increasing antioxidant enzyme activity. TAU could prevent lipid peroxidation in rabbit sperm (Alvarez & Storey, 1983). TAU reversed SOD activity in oxidative stress induced by cadmium or arsenic (Sinha *et al*., 2008; Das *et al*., 2009). Higuchi *et al*. (2012) found that TAU enhanced SOD activity in the eel testicular tissue. TAU prevented apoptosis and oxidative stress induced by doxorubicin in rat testicles (Das *et al*., 2012). Reducing cell death by TAU may relate to inhibition of oxidative stress in TM3 cells. ROS generation can stimulate apoptosis signaling pathways (Redza-Dutordoir & Averill-Bates, 2016). Oxidative stress can induce apoptosis in Leydig cells (Wang *et al*., 2019).

## CONCLUSIONS

In summary, this study showed that TAU induces autophagy and suppresses apoptosis in TM3 cells. This increasing autophagy is accompanied by increasing testosterone levels and reducing oxidative stress.
